# Post-endoscopic retrograde cholangiopancreatography cholangitis after endoscopic treatment of post-transplant biliary strictures: a retrospective study

**DOI:** 10.1186/s12893-025-03106-1

**Published:** 2025-08-09

**Authors:** Chengcheng Christine Zhang, Ronald Koschny, Christian Rupp, Patrick Michl, Arianeb Mehrabi, Cyrill Wehling, Marcus Kantowski, Peter Sauer

**Affiliations:** 1 Department of Gastroenterology, University Hospital Heidelberg, Im Neuenheimer Feld 410, 69120 Heidelberg, Germany; 2 Department of General, Visceral and Transplantation Surgery, University Hospital Heidelberg, Im Neuenheimer Feld 420, 69120 Heidelberg, Germany; 3 Department of Gastroenterology, Helios Klinikum Pforzheim, Kanzlerstraße 2-6, 75175 Pforzheim, Germany; 4 Interdisciplinary Center of Endoscopy, University Hospital Heidelberg, Im Neuenheimer Feld 410, 69120 Heidelberg, Germany

**Keywords:** Cholangitis, Cholestasis, Endoscopic retrograde cholangiopancreatography, Liver transplantation, Postoperative complications

## Abstract

**Background and aim:**

Biliary strictures after liver transplantation are associated with significant morbidity and mortality. Endoscopic retrograde cholangiopancreatography (ERCP) is the preferred approach. Post-ERCP cholangitis is a complication of this procedure. We aimed to evaluate the incidence of post-ERCP cholangitis in patients with post-transplant biliary strictures, their impact on survival, and identify potential risk factors.

**Methods:**

This retrospective study evaluated liver transplant recipients with biliary strictures treated with balloon dilatation at defined intervals. Primary clinical endpoints were the incidence of post-ERCP cholangitis, overall survival, and identification of potential risk factors.

**Results:**

Two hundred patients with a median follow-up period of 6 years (IQR 2–10 years) were included. Anastomotic and non-anastomotic strictures were diagnosed in 132 and 68 patients, respectively. Overall, 930 ERCP procedures were performed, and post-ERCP cholangitis was detected in 148 procedures (15.9%). Patients with post-ERCP cholangitis showed significantly worse overall survival rates (median, 9 vs. 15 years; log-rank test, *p* < 0.001), were significantly more frequently diagnosed with non-anastomotic strictures (44.6% vs. 25%; *p* = 0.004), and had significantly higher treatment failure rates (*n* = 24/92; 26.1% vs. *n* = 13/108; 12%; *p* = 0.02) compared to those without cholangitis. Independent risk factors for cholangitis included the presence of non-anastomotic strictures (OR 3.1), and first-time ERCP intervention with sphincterotomy (OR 6.31).

**Conclusions:**

Post-ERCP cholangitis is a relevant complication of endoscopic treatment and is associated with the presence of non-anastomotic strictures and higher treatment failure rates. Since the success rate of endoscopic intervention in these complex strictures is limited, an optimized peri-interventional management and tailored antibiotic therapy may become particularly important for the further treatment and prognosis of these patients.

**Supplementary Information:**

The online version contains supplementary material available at 10.1186/s12893-025-03106-1.

## Introduction

Biliary complications occur in approximately 5–25% of patients after orthotopic liver transplantation and are associated with high morbidity and mortality [1, 2]. Biliary strictures represent the most common complication with an overall incidence of 4−20% [3, 4], and are classified as anastomotic or non-anastomotic strictures [5, 6]. Interventional management via endoscopic retrograde cholangiopancreatography (ERCP) is the preferred approach for the treatment of biliary post-transplant complications. The different endoscopic therapeutic strategies comprise balloon dilatation and/or plastic stent and self-expanding metal stent (SEMS) placement [4, 5, 7–11]. Nevertheless, a consensus on the optimal therapeutic endoscopic regimen does not exist. A recent study showed that scheduled endoscopic dilatation of post-transplant anastomotic and non-anastomotic strictures was effective and safe, with high success rates depending on the type of biliary strictures, while worse prognosis and higher treatment failure rates occurred in patients with non-anastomotic strictures [12]. This therapeutic regimen could control the symptoms of the patients with a significant improvement in overall survival.

ERCP is an invasive procedure associated with several procedure-related complications, such as pancreatitis, bleeding, cholangitis, and perforation [13]. The reported post-ERCP complication rates for the general population range from 3 to 15% [14, 15]. However, data on post-ERCP complications in liver transplant (LT) recipients are scarce, although some studies have shown similar incidences of overall ERCP complication rates (2–18%), including bleeding, pancreatitis, and cholangitis in LT recipients, compared to the general population [2, 16, 17]. Nevertheless, data regarding the incidence and specific risk factors for the development of post-ERCP cholangitis in this patient population are lacking, which is important considering the use of immunosuppressive therapy in these patients. Besides procedure-related factors, patient-related factors in this special population (e.g., immunosuppressive therapy) might also play an important role in the development of these complications and might have an impact on the outcomes of ERCP and therapeutic strategies for these patients. Therefore, this retrospective study aimed to assess the incidence of post-ERCP cholangitis in patients with post-transplant biliary strictures, evaluate the impact on survival, and identify potential risk factors to improve peri-interventional management.

## Methods

### Study design and population

This retrospective study was conducted at a tertiary referral center for liver transplantation between 2000 and 2019. All patients received liver grafts after brain death of the donors. Patients with suspected biliary strictures, defined as dilatation of the intra- or extrahepatic biliary system on imaging in association with elevated cholestatic laboratory parameters, and clinical signs of jaundice, cholangitis, or pruritus, were considered for endoscopic diagnosis and treatment, as previously described [12]. Patients were followed up until November 2023 from the time of the first therapeutic intervention until the last contact or death, with a median follow-up period of 6 years (IQR 2–10 years).

As previously described [12], an anastomotic stricture was defined as a stricture localized at or adjacent to the biliary anastomosis. Non-anastomotic strictures were defined as strictures with involvement of the donor hepatic bile duct up to 0.5 cm proximal to the biliary anastomosis and/or intrahepatic biliary strictures [4]. Patients with choledochojejunostomy were excluded. According to the Tokyo guidelines [18], the diagnostic criteria for acute cholangitis are systemic inflammation, cholestasis, and bile duct lesions (based on imaging findings). Acute cholangitis was further graded into three severity classes: grades I, II, and III.

Laboratory results were obtained from all patients before and after the ERCP procedure, and clinical signs of cholangitis, including fever, right upper quadrant pain, and jaundice, were recorded before and after ERCPs were performed. Post-ERCP cholangitis was defined as the onset of new cholangitis after endoscopic intervention or the worsening of characteristic clinical symptoms and abnormal laboratory results within 1 to 3 days. Complications were recorded from the time of ERCP until discharge. After discharge, patients were instructed to call or return to the hospital if they experienced any issues (e.g., abdominal pain, fever, melena, or hematemesis). The patients were closely followed up in the liver transplantation outpatient clinic at regular intervals. All patients with post-ERCP cholangitis were treated with antibiotics based on microbiological testing, if available. Patients with clinical signs of cholangitis before ERCP were excluded from the study.

### Endoscopic treatment

ERCP was performed as previously described [19]. A biliary stricture was confirmed after the application of a contrast medium under fluoroscopy. Endoscopic treatment consisted of sphincterotomy and balloon dilation using a 12-, 18-, or 24-Fr balloon dilation catheter depending on the stenosis grade (MaxForce, Boston-Scientific, Marlborough, MA, USA). In some cases, additional short-term placement of a plastic stent (10 Fr) or temporary placement of a fully covered SEMS (Wallstent, Boston-Scientific, Marlborough, MA, USA; Hanarostent, Olympus, Tokyo, Japan) was performed at the discretion of the endoscopist. Both the anatomy and clinical failure of the initial treatment were considered when deciding whether a plastic stent or a SEMS was placed. Usually, SEMS insertion is preferred for anastomotic strictures, whereas plastic stents are used for endoscopically reachable non-anastomotic strictures.

The scheduled endoscopic dilatation program was performed as previously described [12]. Briefly, scheduled dilatation treatment was conducted 4 weeks after the first procedure, followed by further dilatations after 3 and 6 months, until morphological resolution of the stricture and clinical improvement of the patients were observed. In cases of acute cholangitis, worsening cholestasis, or other clinical symptoms, ERCP was performed independently of the protocol. As previously described [19], sustained clinical success was defined as the absence of further endoscopic interventions for ≥ 3 months after completion of the initial endoscopic therapy, normalized laboratory parameters, and absence of clinical symptoms related to cholangitis or jaundice. The need for a definitive alternative treatment to endoscopy was defined as treatment failure (i.e., re-transplantation, percutaneous transhepatic drainage, and/or hepaticojejunostomy). Both recurrent cholangitis and persistent stricture may lead to the decision to declare treatment failure. Biliary stricture recurrence was defined as the occurrence of a biliary stricture after sustained clinical success.

All endoscopic interventions were performed with a single-shot peri-interventional antibiotic prophylaxis (ampicillin/sulbactam or adaptive therapy, based on bile culture results, when available), and ciprofloxacin was used in cases of a known penicillin allergy. Candida infections were treated with fluconazole.

### Bile sampling and microbiological analysis

Bile sampling and microbiological analyses were performed as described previously [20, 21]. Briefly, bile samples were obtained after cannulation, before the therapeutic procedure was performed. All biliary samples were placed in sterile tubes, delivered to the microbiology laboratory within 2 h of collection, and cultured according to the standard laboratory protocols. Routine antibiotic susceptibility tests were performed.

### Primary and secondary endpoints

The primary endpoints of this study were the incidence of post-ERCP cholangitis after scheduled endoscopic interventions in patients with post-transplant biliary strictures, overall survival, and potential risk factors for post-ERCP cholangitis after liver transplantation. Secondary endpoints were the incidence of more than one post-ERCP cholangitis episode, type of biliary stricture, sustained clinical success, treatment failure, peri-interventional antibiotic prophylaxis, microbiological analysis of bile fluid, immunosuppressive therapy, need for intensive care unit (ICU) treatment, and hospitalization duration.

### Statistical analysis

Continuous and categorical data were compared using the non-parametric Wilcoxon rank-sum test and χ^2^ or Fisher’s exact test, respectively. To predict the risk of developing post-ERCP cholangitis, the binary logistic regression method was used. Covariates that showed significant results in the univariate analysis were further analyzed in multivariate analysis. Independent prognostic factors for survival were identified using multivariate Cox regression analysis with simultaneous adjustment for type of biliary strictures, and occurrence of post-ERCP cholangitis. A *p* < 0.05 was considered statistically significant. Kaplan−Meier product limit estimator was used to estimate the overall survival. The log-rank test was used to calculate differences in actuarial survival. Statistical significance was set at *p* < 0.05. All analyses were performed using the SPSS version 25 software (IBM Corp., Armonk, NY, USA).

### Consent

Written informed consent was obtained from all patients, and consent for data acquisition and analysis was obtained. The study protocol, data acquisition, and evaluation were approved by the local ethics committee of Heidelberg University (Heidelberg, Germany) (approval number S-043/2011). This study was conducted in accordance with the 1975 Declaration of Helsinki (6th Revision, 2008).

## Results

### Baseline characteristics

Between 2000 and 2019, 200 patients who underwent orthotopic liver transplantation were diagnosed with either biliary anastomotic strictures (*n* = 132) or non-anastomotic strictures (*n* = 68) and referred for endoscopic treatment at the University Hospital Heidelberg (Table 1). The median follow-up period was 6 years (IQR: 2–10 years).

**Table 1:** Baseline characteristics

Overall patients, *n*	200
Sex (male/%)	151 (75.5)
Age at diagnosis, (years) mean ± SD	52 ± 12
Etiology of liver disease (n)	
Alcohol	50
Virus	67
HCC	60
PSC	10
Other	69
Biliary dilatation on imaging, n (%)	171 (85.5)
Median duration from transplant to biliary stenosis, months	7
Type of biliary stenosis	
Anastomotic stricture, n (%)	132 (66)
Non-anastomotic stricture, n (%)	68 (34)
Overall ERCP procedures, n	930
Median number of endoscopic interventions, n [IQR]	4 [3–6]
Number of post-ERCP cholangitis, n (%)	148 (15.9)
Cholangitis severity grade	
Grade I	30 (20.3)
Grade II	111 (75)
Grade III	7 (4.7)
Patients with post-ERCP cholangitis, n (%)	92 (46)
Patients without cholangitis, n (%)	108 (54)
Median number of post-ERCP cholangitis episodes, n [IQR]	1 [1–2]
Other ERCP complications	
Pancreatitis, n (%)	12 (6)
Bleeding, n (%)	1 (0.5)
Perforation, n (%)	4 (2)
Median follow-up, years [IQR]	6 [2–10]

SD, standard deviation; IQR, interquartile range; HCC, hepatocellular carcinoma; ERCP, endoscopic retrograde cholangiopancreatography

The final study cohort comprised 200 patients (151 men and 49 women), with a mean age of 52 years (range 16–70 years). A total of 930 ERCP procedures were performed in these 200 patients, with a median of four endoscopic interventions per patient (IQR: 3–6). A total of 148 cholangitis episodes were detected among the 930 ERCP procedures performed. The post-ERCP cholangitis rate was 15.9% per procedure (*n* = 148/930). Of the 148 post-ERCP cholangitis episodes, 39 (20.3%) were classified as Grade I (mild), 111 (75%) as Grade II (moderate), and 7 (4.7%) as Grade III (severe), with no cholangitis-related mortalities. 92 patients had at least one cholangitis episode (*n* = 92/200; 46%), while 108 patients did not develop post-ERCP cholangitis (*n* = 108/200; 54%). The median number of post-ERCP cholangitis episodes was one per patient (IQR: 1–2).

Further ERCP-related complications were post-ERCP pancreatitis (*n* = 12/200; 6%), bleeding (*n* = 1/200; 0.5%), and bile duct perforation (*n* = 4/200; 2%). All complications were controlled by conservative measures, and no patient died because of endoscopic procedure-related adverse events.

### Characteristics of patients with and without post-ERCP cholangitis

**Fig. 1:**
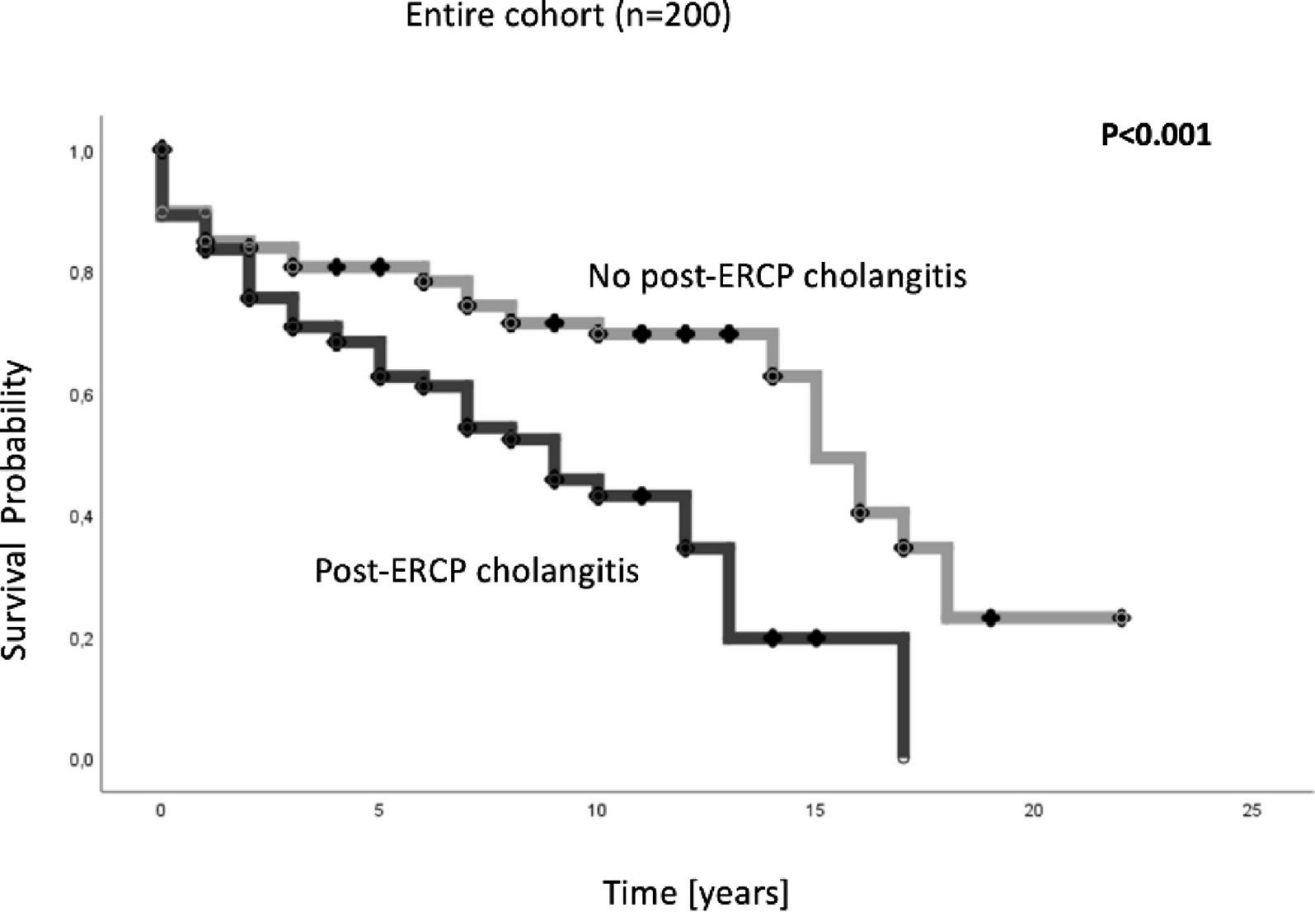
Overall survival rates of patients with post-ERCP cholangitis compared to patients without post-ERCP cholangitis. Kaplan−Meier analysis revealed a reduced median overall survival rate in patients with post-ERCP cholangitis compared to that in patients without post-ERCP cholangitis (9 vs. 15 years; log-rank test: *p* < 0.001). ERCP, endoscopic retrograde cholangiopancreatography

At the last follow-up, 114 patients (57%) were alive, 78 (39%) had died, and 8 were lost to follow-up (4%). The median survival for the entire cohort was 13 (95% CI 10.7–15.3) years (shown in Fig. 1). Patients with post-ERCP cholangitis had significantly lower overall survival rates with a median overall survival of 9 years (95% CI: 6.3–11.7) compared to patients without post-ERCP cholangitis with a median overall survival of 15 years (95% CI: 13.5–16.5) (log-rank: *p* < 0.001).

Of the 200 patients, 107 (53.5%) received immunosuppressive treatment with tacrolimus, 74 (37%) were treated with cyclosporine, and 122 (61%) received combined immunosuppressive therapy (Table 2). No significant difference was observed between the patients with and without post-ERCP cholangitis. Patients who received peri-interventional antibiotic prophylaxis comprised 96% (*n* = 192/200) of the cohort. Microbiological analysis revealed a significantly higher rate of detection of bacteria (82.6% vs. 14.8%; *p* = 0.001) and fungi (28.3% vs. 0.9%; *p* = 0.001) in the bile fluid of patients with post-ERCP cholangitis compared to those without it. No difference was observed in the detection of multidrug-resistant bacteria in either group and in the history of bacteria- or fungi-positive bile. The median duration of hospitalization was significantly higher in patients with post-ERCP cholangitis than in those without it (6 days [IQR: 4–11] vs. 2 days [IQR: 2–2]; *p* = 0.001), although no difference was observed in the need for ICU treatment.

**Table 2:** Characteristics of patients with post-ERCP cholangitis and without post-ERCP cholangitis

	Overall patients (*n* = 200)	Post-ERCP cholangitis (*n* = 92)	No post-ERCP cholangitis (*n* = 108)	*p*-value
Sex (male/%)	151 (75.5)	73 (79.3)	78 (72.2)	0.25
Age at diagnosis, (years) mean ± SD	52 ± 12	53 ± 12	52 ± 13	0.26
Immunosuppressive therapy				
Tacrolimus, n (%)	107 (53.5)	44 (47.8)	63 (58.3)	0.14
Cyclosporin, n (%)	74 (37)	36 (39.1)	38 (35.2)	0.57
Everolimus, n (%)	5 (2.5)	5 (5.4)	0 (0)	0.1
Sirolimus, n (%)	4 (2)	3 (3.3)	1 (0.9)	0.24
Steroids, n (%)	36 (18)	20 (21.7)	16 (14.8)	0.21
MMF, n (%)	106 (53)	49 (53.3)	57 (52.8)	0.95
Combined immunosuppressive therapy, n (%)	122 (61)	56 (60.9)	66 (61.1)	0.97
Peri-interventional antibiotic prophylaxis, n (%)	192 (96)	92 (100)	100 (92.6)	0.08
Microbiological analysis of the bile fluid				
Bacteria, n (%)	92 (46)	76 (82.6)	16 (14.8)	**0.001**
Fungi, n (%)	27 (13.5)	26 (28.3)	1 (0.9)	**0.001**
Multiresistant bacteria, n (%)	17 (18.1)	13 (16.3)	4 (28.6)	0.27
History of bacteria- or fungi-positive bile, n (%)	95 (47.5)	41 (44.6)	54 (50)	0.44
ICU treatment, n (%)	8 (4)	2 (2.2)	6 (5.6)	0.22
Median hospitalization time, days [IQR]	3 [2–7]	6 [4–11]	2 [2–2]	**0.001**
Re-transplantation, n (%)	23 (11.5)	12 (13)	11 (10.2)	0.66
Death, n (%)	78 (39)	39 (42.4)	39 (36.1)	0.39

SD, standard deviation; IQR, interquartile range; MMF, mycophenolate mofetil; ICU, intensive care unit; ERCP, endoscopic retrograde cholangiopancreatography

Bold values indicate significant *p*-values (< 0.05)

### Characteristics of patients with single vs. multiple post-ERCP cholangitis episodes

Detailed characteristics of the patients with single vs. multiple post-ERCP cholangitis episodes are shown in Additional file Table 1. The median number of post-ERCP cholangitis episodes was one (IQR: 1–2). Twenty-six patients (23 men; mean age, 53 years) were diagnosed with more than one episode of cholangitis. Patients with multiple post-ERCP cholangitis episodes were significantly more often on combined immunosuppressive therapy compared to patients with a single post-ERCP cholangitis episode (*n* = 22/26, 84.6% vs. *n* = 33/66, 50%; *p* < 0.001; Additional file Table 1).

### Endoscopic interventions

All patients underwent scheduled endoscopic therapy for biliary post-transplant anastomotic strictures (*n* = 132/200; 66%) and non-anastomotic strictures (*n* = 68/200, 34%; Table 3). Balloon dilation was the primary therapy for all patients. Based on the clinical presentation, laboratory results, or morphological findings, the examiners decided whether other treatment methods were required in addition to balloon dilatation. This subsequent therapy comprised additional placement of plastic stents and SEMS.

**Table 3:** Endoscopic intervention data of patients with post-ERCP cholangitis and without post-ERCP cholangitis

	Overall patients (*n* = 200)	Post-ERCP cholangitis (*n* = 92)	No post-ERCP cholangitis (*n* = 108)	*P*-value
Type of biliary stenosis				
Anastomotic stricture, n (%)	132 (66)	51 (55.4)	81 (75)	**0.004**
Non-anastomotic stricture, n (%)	68 (34)	41 (44.6)	27 (25)	**0.004**
First ERCP with sphincterotomy, n (%)	23 (11.5)	18 (19.6)	5 (4.6)	**0.001**
Repeated ERCP, n (%)	178 (89)	75 (81.5)	103 (95.4)	**0.002**
Endoscopic intervention before cholangitis				
Balloon dilatation, n (%)	148 (74)	73 (79.3)	75 (69.4)	0.15
Plastic stent, n (%)	32 (16)	10 (10.9)	22 (20.4)	0.18
SEMS placement, n (%)	11 (5.5)	1 (1.1)	10 (9.3)	0.23
Rendezvous procedure, n (%)	6 (3)	3 (3.3)	3 (2.8)	0.42
Sustained clinical success, n (%)	150 (75)	64 (69.6)	86 (79.6)	0.11
Median time interval to sustained success, months, [IQR]	9 [6–13]	8 [6–12]	9 [6–14]	0.92
Dropout before achievement of any endpoint, n (%)	13 (6.5)	4 (4.3)	9 (8.3%)	0.56
Treatment failure, n (%)	37 (18.5)	24 (26.1)	13 (12)	**0.02**
Recurrence of biliary strictures, n (%)	37 (18.5)	22 (23.9)	15 (13.9)	0.1
Median time interval to recurrence, months	7 [3–16]	6 [3–17]	8 [3.5–15]	0.92
Successful treatment of recurrent stricture, n (%)	29 (76.3)	18 (78.3)	11 (73.3)	0.73
Median number of endoscopic interventions, n [IQR]	4 [3–6]	5 [4–8]	4 [3–5]	0.33

IQR, interquartile range; ERCP, endoscopic retrograde cholangiopancreatography; SEMS, self-expanding metal stent

Bold values indicate significant *p* -values (< 0.05)

Patients with post-ERCP cholangitis were significantly more frequently diagnosed with non-anastomotic strictures compared to those without it (*n* = 41/92, 44.6% vs. *n* = 27/108, 25%; *p* = 0.004). Post-ERCP cholangitis occurred more frequently after the first-time ERCP intervention with sphincterotomy (*n* = 18/92, 19.6% vs. *n* = 5/108, 4.6%; *p* = 0.001), whereas follow-up ERCP interventions were performed more often in patients without post-ERCP cholangitis (*n* = 103/108, 95.4% vs. *n* = 75/92, 81.5%; *p* = 0.002). Endoscopic interventions included balloon dilation, placement of plastic stents and SEMS, and rendezvous procedure. No significant difference was observed in the type of endoscopic intervention between the groups. Sustained clinical success rate of endoscopic treatment was 75% (*n* = 150/200) in all patients: 69.6% in those with post-ERCP cholangitis (*n* = 64/92) and 79.6% in patients without post-ERCP cholangitis (*p* = 0.11). The median time interval to sustained clinical success was 9 (IQR: 6–13) months and did not differ between the groups. Significantly higher treatment failure rates were detected in patients with post-ERCP cholangitis compared to patients without post-ERCP cholangitis (*n* = 24/92; 26.1% vs. *n* = 13/108; 12%; *p* = 0.02). Recurrence of biliary strictures was diagnosed in 18.5% patients (*n* = 37/200), and 76% patients (*n* = 29/37) with recurrent biliary strictures could be re-treated successfully by endoscopic interventions. The median number of endoscopic interventions was 4 (IQR: 3–6) and did not differ significantly between the groups.

### Characteristics of patients with anastomotic and non-anastomotic strictures depending on the occurrence of post-ERCP cholangitis

**Fig. 2:**
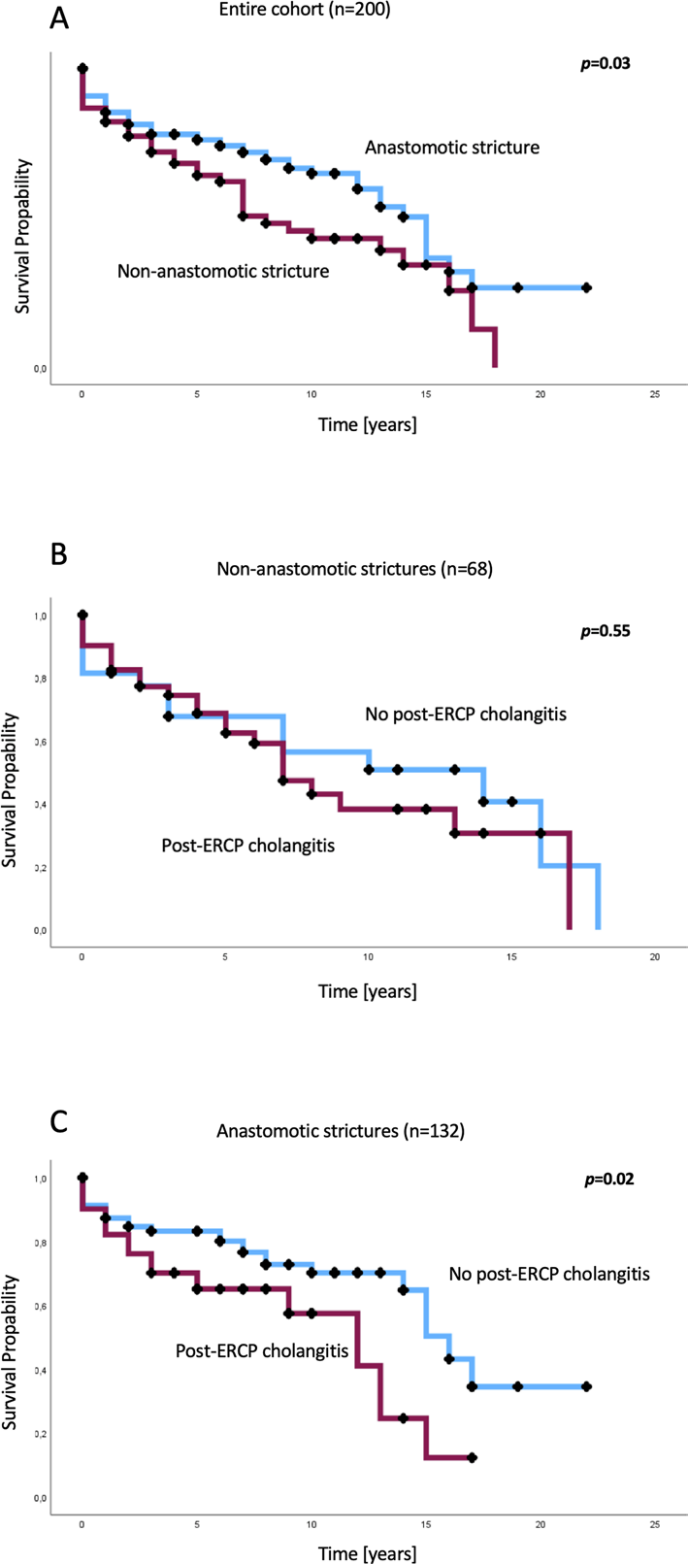
Overall survival rates of patients with anastomotic and non-anastomotic strictures depending on the occurrence of post-ERCP cholangitis. Kaplan−Meier analysis showed a significantly higher median overall survival rate in patients with anastomotic strictures than that in patients with non-anastomotic strictures (15 vs. 8 years; log-rank test: *p* = 0.03) (**A**). Although not statistically significant, further subgroup analysis in patients with non-anastomotic strictures revealed a reduced median overall survival rate in patients with post-ERCP cholangitis (7 years) compared to patients without post-ERCP cholangitis (14 years) (log-rank test: *p* = 0.55) (**B**), whereas the development of post-ERCP cholangitis in patients with anastomotic strictures resulted in significantly shorter overall survival rates (12 years vs. 16 years; log-rank test: *p* = 0.02) (**C**). ERCP, endoscopic retrograde cholangiopancreatography

As shown in Fig. 2A, patients with anastomotic strictures had significantly higher overall survival rates, with a median overall survival of 15 (95% CI: 13–17) years, compared to patients with non-anastomotic strictures, with a median overall survival of 8 (95% CI: 5–11) years (log-rank test: *p* = 0.03). Although not statistically significant, the median overall survival rates in patients with non-anastomotic strictures who developed post-ERCP cholangitis were lower at 7 (95% CI: 5–9) years, compared to patients with non-anastomotic strictures without post-ERCP cholangitis, with a median overall survival of 14 (95% CI: 3–25) years (log-rank test: *p* = 0.55; Fig. 2B). Among patients with anastomotic strictures, those with post-ERCP cholangitis showed significantly lower overall survival rates, with a median overall survival of 12 (95% CI: 8–16) years, compared to those without post-ERCP cholangitis, with a median overall survival of 16 (95% CI: 14–18) years (log-rank test: *p* = 0.02; Fig. 2C).

Detailed characteristics of patients with anastomotic and non-anastomotic strictures depending on the occurrence of post-ERCP cholangitis are shown in Additional file Table 2.

### Factors associated with cholangitis and outcomes

As shown in Table 4, the univariate regression analysis identified non-anastomotic strictures (OR 3.1; *p* = 0.001), and first-time ERCP intervention with sphincterotomy (OR 6.31; *p* < 0.001) as risk factors for the occurrence of post-ERCP cholangitis. Multivariate analysis confirmed the presence of non-anastomotic strictures (OR 2.66; *p* = 0.002) and first-time ERCP intervention with sphincterotomy (OR 5.36; *p* = 0.002) as independent risk factors for cholangitis.

**Table 4:** Univariate and multivariate regression analysis

Risk factor	Univariate		Multivariate	
	OR (95% CI)	*p*-value	OR (95% CI)	*p*-value
Sex (male)	0.69 (0.35–1.34)	0.27		
Age at diagnosis (years)	1.01 (0.98–1.03)	0.73		
Immunosuppressive therapy				
Tacrolimus, n (%)	0.83 (0.2–3.5)	0.81		
Cyclosporin, n (%)	1.3 (0.31–5.71)	0.69		
Sirolimus, n (%)	6.13 (0.4–92.8)	0.19		
Steroids, n (%)	1.24 (0.54–2.86)	0.61		
MMF, n (%)	1.12 (0.51–2.42)	0.78		
Combined immunosuppressive therapy, n (%)	0.94 (0.43–2.09)	0.88		
First ERCP with sphincterotomy	6.31 (2.08–19.15)	**0.001**	5.36 (1.85–15.49)	**0.002**
Non-anastomotic stricture	3.1 (1.6–5.97)	< **0.001**	2.66 (1.43–4.92)	**0.002**

CI, confidence interval; ERCP, endoscopic retrograde cholangiopancreatography; MMF, mycophenolate mofetil; OR: odds ratio

Bold values indicate significant *p*-values (< 0.05)

To determine factors that might influence survival, multivariate cox regression analyses were performed. This analysis demonstrated that the presence of non-anastomotic strictures was significantly associated with reduced survival (OR 1.58; *p* = 0.04), while the occurrence of post-ERCP cholangitis was not (Additional file Table 3).

## Discussion

Cholangitis is a serious complication of biliary obstruction, which requires adequate drainage and antibiotic therapy [22, 23]. Infections and biliary complications after transplantation are a significant cause of morbidity and mortality, which could worsen the condition of the LT recipients [24]. To the best of our knowledge, this is the first study to evaluate the incidence and impact of post-ERCP cholangitis after endoscopic interventions in patients with post-transplant biliary strictures and to identify potential risk factors to optimize the peri-interventional management in immunosuppressed patients. Although stenting or the combination of balloon dilatation and stenting is recommended as the therapy of choice for treatment of biliary strictures [9], some studies have shown higher risks of acute and chronic bacterial cholangitis, as well as higher occlusion rates for biliary strictures, especially when using plastic stents [19, 25–31]. In addition, available publications reporting on balloon dilatation alone are mostly based on very small study cohorts or case reports with heterogeneous study designs. However, the optimal technique in this context remains debatable. At our institution, we implemented a strategy of scheduled endoscopic treatment for biliary post-transplant strictures, which could control the symptoms of the patients and improve survival [12]. Although this concept shows advantages in these patients, the potential complications of the procedure must be weighed against its benefits. Therefore, the cholangitis rate of 15.9% per procedure observed in our study should be considered carefully. This incidence is higher than that reported in other studies focusing on LT recipients, with cholangitis rates ranging from 3.3 to 5.3% [2, 16, 32–34]. However, direct comparison is difficult because most previous studies consisted of heterogeneous, small cohorts with different etiologies and indications for ERCP procedures, and various definitions of cholangitis. All ERCP procedures conducted in this study cohort were therapeutically intended, whereas other studies also included diagnostic ERCP procedures in their analysis [34]. Therapeutically intended ERCP procedures may carry a higher risk of procedure-related complications, particularly post-ERCP cholangitis, as the time of intervention is often prolonged and the risk of ascending cholangitis by repeated manipulation might be higher. A recent study could identify ischemic type biliary lesions as independent a risk factor for infections following ERCP and reported high infection rates post-ERCP of more than 60% in these patients [35]. Therefore, the high cholangitis rate observed in this study can likely be attributed to the significant proportion (34%) of patients diagnosed with the more unfavorable non-anastomotic strictures. It seems reasonable to assume that a more complex biliary situation possibly requiring more intricate intervention strategies, may increase the risk of infections and post-ERCP cholangitis. In line with this, cholangitis rates after ERCP, particularly for non-anastomotic strictures, are reported to be high, ranging from 33 to 74% [31, 35, 36]. This could be attributed to the presence of multiple intrahepatic strictures within the bile duct system and cast formation in patients with non-anastomotic strictures, which may impede complete biliary drainage after ERCP and predispose for colonization of microorganisms [35]. Furthermore, impaired biliary drainage after the injection of contrast media into obstructed bile ducts is a key risk factor for the development of post-ERCP infections [37].

Peri-procedural antibiotic administration is indicated if appropriate biliary drainage is not obtained. Immunosuppressed patients, as well as those after liver transplantation, also have a higher risk of developing post-ERCP biliary infections [38]. Various studies have shown that the risk of acute infections in liver transplant recipients on long-term immunosuppressive therapy is increased [24, 39]. Consequently, nearly almost all our patients received peri-interventional antibiotic prophylaxis to reduce the risk or were already undergoing antibiotic therapy, as recommended by the European Society of Gastrointestinal Endoscopy and the American College of Gastroenterology [9, 40].

Patients with post-ERCP cholangitis had significantly lower overall survival rates than those without cholangitis. However, this observed association between cholangitis and reduced survival cannot be solely attributed to the occurrence of post-ERCP cholangitis. Patients with post-ERCP cholangitis were also significantly more frequently diagnosed with non-anastomotic strictures compared to patients without post-ERCP cholangitis and showed higher rates of treatment failure. Moreover, non-anastomotic strictures were identified as independent risk factor for the occurrence of post-ERCP cholangitis. The patients with non-anastomotic strictures have worse prognosis in terms of successful treatment, graft survival, and overall survival compared to those with anastomotic strictures [12]. Our current results show that this subgroup, with the more complex biliary lesions and lower success rate of endoscopic therapy, also experiences a higher incidence of post-interventional cholangitis. The presence of non-anastomotic strictures was significantly associated with worse outcomes in this study, while the occurrence of post-ERCP cholangitis could not be identified as an independent risk factor for survival. Thus, the occurrence of post-ERCP cholangitis appears to be more likely a marker of a more severe disease in patients with non-anastomotic strictures and complex biliary conditions, rather than an independent driver of mortality. In addition, microbiological analysis of the bile fluid of patients with non-anastomotic strictures revealed significantly higher rates of bacteria and fungi compared to those in patients with anastomotic strictures. Furthermore, patients with post-ERCP cholangitis had higher rates of detection of bacteria and fungi in the bile fluid compared to those without it. These findings suggest that patients may already harbor bacteria/fungi in the bile asymptomatically, and the endoscopic manipulation then precipitates symptomatic cholangitis.

Another important clinical observation is that patients on combined immunosuppressive therapy were significantly more likely to experience multiple episodes of cholangitis. The heavier immunosuppression may predispose these patients to recurrent infections. Although this is only an association observed in a subgroup, this finding should be considered in the care management of these patients, as it may warrant closer monitoring or the need for antibiotic prophylaxis.

Notably, almost all patients in this study cohort received peri-interventional antibiotic prophylaxis. Nevertheless, cholangitis still occurred in approximately one out of every six ERCPs. This suggests that the current prophylactic antibiotic regimen (usually a single-shot dose) may be insufficient in high-risk scenarios (e.g. non-anastomotic strictures). Another important point in this context is the timing of the antibiotic administration. Usually, antibiotic prophylaxis is administered during examinations at our center. However, a recent study identified delayed or post-ERCP antibiotic administration as a risk factor for infections [35]. Consequently, the optimized peri-interventional management in these patients could be a prophylactic antibiotic treatment before endoscopic intervention to reduce the incidence of post-ERCP cholangitis. Furthermore, this observation may support extending the antibiotic treatment (beyond a single prophylactic dose), especially in patients with non-anastomotic strictures. As this subgroup with non-anastomotic strictures do not only have poorer therapeutic outcomes but also higher infectious complication rates, the need for special care in these patients is crucial.

Biliary tract infections play an important role in bile duct destruction [41], and successful treatment of biliary post-transplant strictures is crucial to prevent the risk of chronic biliary obstructions with development of chronic cholangitis and graft failure [42]. Therefore, microbiological testing is important for an improved specific antibiotic treatment of cholangitis in these patients by individually adapted bile culture antibiogram. Besides a tailored and more aggressive antibiotic strategy, the timing of antibiotic prophylaxis with early pre-interventional administration seems to be crucial for patient outcomes. Altogether, the development of post-ERCP cholangitis may affect survival and emphasizes the need for adequate antibiotic therapy, regardless of whether the worse prognosis can be attributed to cholangitis or the presence of less favorable non-anastomotic strictures.

Regarding the scheduled endoscopic therapy regimen for biliary post-transplant strictures at our center, a sustained success rate of 75% for the entire patient cohort was detected in the present study. Treatment failure of the biliary strictures occurred in 18.5% patients in the entire cohort. This is in line with the previously published data [12] as well as reports on the success rates of other studies evaluating the endoscopic treatment of biliary post-transplant strictures [7, 10, 19, 31, 43–50]. The rate of other ERCP complications (pancreatitis, bleeding, perforation) in our study was similar to that reported in other studies [7, 8, 19, 31, 43, 48]. All complications were minor and were manageable by conservative measures. The outcome after scheduled endoscopic treatment of biliary post-transplant strictures regarding sustained success and survival, and the finding that most post-ERCP complications including cholangitis were mild to moderate without severe or fatal complications justify this specific therapeutic regimen.

Nonetheless, this study has several limitations. First, it was a retrospective, not controlled and/or randomized study, which may have reduced the validity of the results. The retrospective, single-center design limits the generalizability of the findings to other centers with different protocols. Additionally, there was no control group for the management strategies regarding the endoscopic treatment or the antibiotic prophylaxis regimen, meaning that superiority over alternatives cannot be demonstrated. Due to the limited number of patients included, certain patient-specific factors, such as comorbidities, were not thoroughly examined, and some potential influences in this context were therefore not evaluated. Future studies with larger sample sizes or multi-center data are needed to identify significant risk factors. Furthermore, the inclusion period spans nearly 20 years, during which endoscopic techniques, immunosuppressants, immunosuppressive protocols, and prophylactic antibiotic strategies have evolved. While the scheduled endoscopic treatment with balloon dilation as first-line therapy for biliary post-transplant strictures remained consistent throughout the period, other evolving factors may have confounded the study’s outcomes. Finally, due to the retrospective design, establishing causality is not possible.

Nevertheless, to the best of our knowledge, this is the largest study cohort with a long follow-up period to evaluate the incidence of post-ERCP cholangitis after endoscopic treatment for biliary strictures following orthotopic liver transplantation. However, future prospective controlled interventional studies focusing on extended or tailored antibiotic prophylaxis to reduce post-ERCP infections are necessary to validate these findings.

In conclusion, post-ERCP cholangitis is a significant complication of the endoscopic treatment of biliary post-transplant strictures. The occurrence of post-ERCP cholangitis appears to be associated with the presence of non-anastomotic strictures and higher treatment-failure rates. Although episodes of severe post-ERCP cholangitis are rare, the occurrence of post-ERCP cholangitis is associated with worse outcomes during long-term follow-up, which may be attributed to the more complex and difficult-to-treat biliary condition in patients with non-anastomotic strictures. These findings underscore the importance of specific antibiotic prophylaxis and culture-guided treatment of cholangitis in such patients to minimize infection risks and to improve long-term prognosis.

## Supplementary Information

Below is the link to the electronic supplementary material.

Supplementary Material 1

## Data Availability

Data availability statement: Data are available on reasonable request from the corresponding author: Chengcheng Christine Zhang, MD, Interdisciplinary Center of Endoscopy, University Hospital Heidelberg, Im Neuenheimer Feld 410, 69120 Heidelberg, Germany.

## Abbreviations

ERCP: Endoscopic retrograde cholangiopancreatography
ICU: Intensive care unit
LT: Liver transplant
SEMS: Self-expanding metal stent
