# Supplementary material for: Post-endoscopic retrograde cholangiopancreatography cholangitis after endoscopic treatment of post-transplant biliary strictures: a retrospective study
Source: BMC Surg. 2025 Aug 9;25:353. doi: 10.1186/s12893-025-03106-1 (PMC12335147; doi:10.1186/s12893-025-03106-1)
Supplement: Supplementary file 1 — Supplementary Material 1 [file 12893_2025_3106_MOESM1_ESM.docx]

**Additional File Table 1: Characteristics of patients with single and multiple post-ERCP cholangitis episodes.**

|  | Overall patients with post-ERCP cholangitis (n=92) | Single post-ERCP cholangitis episode (n=66) | Multiple post-ERCP cholangitis episodes (n=26) | *p*-value |
| --- | --- | --- | --- | --- |
| Sex (male/%) | 73 (79.3) | 50 (75.8) | 23 (88.5) | 0.35 |
| Age at diagnosis, (years) mean ± SD | 53 ± 12 | 52 ± 12 | 53 ± 12 | 0.22 |
| Immunosuppressive therapy |  |  |  |  |
| Tacrolimus, n (%) | 44 (47.8) | 32 (48.5) | 12 (46.2) | 0.88 |
| Cyclosporin, n (%) | 36 (39.1) | 27 (40.9) | 9 (34.6) | 0.87 |
| Everolimus, n (%) | 5 (5.4) | 3 (4.5) | 2 (7.7) | 0.24 |
| Sirolimus, n (%) | 3 (3.3) | 3 (4.5) | 0 (0) | **0.02** |
| Steroids, n (%) | 20 (21.7) | 11 (16.7) | 9 (34.6) | **0.002** |
| MMF, n (%) | 49 (53.3) | 36 (54.5) | 13 (50) | 0.87 |
| Combined immunosuppressive therapy, n (%) | 56 (60.9) | 33 (50) | 22 (84.6) | **< 0.001** |
| Peri-interventional antibiotic prophylaxis, n (%) | 92 (100) | 66 (100) | 26 (100) |  |
| Microbiological analysis of the bile fluid |  |  |  |  |
| Bacteria, n (%) | 76 (82.6) | 54 (81.2) | 22 (84.6) | 0.52 |
| Fungi, n (%) | 26 (28.3) | 17 (25.8) | 9 (34.6) | 0.13 |
| Multiresistant bacteria, n (%) | 13 (16.3) | 9 (13.6) | 4 (15.4) | 0.94 |
| History of bacteria- or fungi-positive bile, n (%) | 41 (44.6) | 30 (45.5) | 11 (42.3) | 0.29 |
| ICU treatment, n (%) | 2 (2.2) | 2 (3) | 0 (0) | 0.07 |
| Median hospitalization time, days [IQR] | 6 [4–11] | 6 [4–11] | 7 [4–9] | 0.51 |
| Severity grade |  |  |  |  |
| Grade I | 23 (25) | 18 (27.3) | 5 (19.2) |  |
| Grade II | 66 (71.7) | 46 (69.7) | 20 (76.9) |  |
| Grade III | 3 (3.2) | 2 (3) | 1 (3.8) |  |
| Median time to cholangitis, days [IQR] | 1 [0–1] | 1 [0–1] | 1 [0–1] | 0.44 |
| Type of biliary stenosis |  |  |  |  |
| Anastomotic stricture, n (%) | 51 (55.4) | 38 (57.6) | 13 (50) | 0.59 |
| Non-anastomotic stricture, n (%) | 41 (44.6) | 28 (42.4) | 13 (50) | 0.44 |
| First ERCP with sphincterotomy, n (%) | 18 (19.6) | 12 (18.2) | 6 (23.1) | 0.31 |
| Repeated ERCP, n (%) | 75 (81.5) | 54 (81.8) | 21 (80.7) | 0.68 |
| Sustained clinical success, n (%) | 64 (69.6) | 47 (71.2) | 17 (65.4) | 0.12 |
| Median time interval to sustained success, months, [IQR] | 8 [6–12] | 8 [6–12] | 7 [6–11] | 0.69 |
| Treatment failure, n (%) | 24 (26.1) | 18 (27.3) | 6 (23.1) | 0.95 |
| Recurrence of biliary strictures, n (%) | 22 (23.9) | 13 (19.7) | 9 (34.6) | **0.01** |
| Median time interval to recurrence, months | 6 [3.25–17.25] | 9 [6–32] | 5 [4–17] | **0.002** |
| Successful treatment of recurrent stricture, n (%) | 18 (78.3) | 11 (84.6) | 7 (77.8) | 0.93 |
| Median number of endoscopic interventions, n [IQR] | 5 [4–8] | 5 [3–7] | 5.5 [4–11] | 0.1 |
| Re-transplantation, n (%) | 12 (13) | 10 (15.1) | 2 (7.7) | 0.06 |
| Death, n (%) | 39 (42.4) | 29 (43.9) | 10 (38.5) | 0.59 |

SD, standard deviation; IQR, interquartile range; MMF, mycophenolate mofetil; ICU, intensive care unit; ERCP, endoscopic retrograde cholangiopancreatography.

Bold values indicate significant *p*-values (< 0.05).

**Additional File Table 2:** **Characteristics of patients with anastomotic and non-anastomotic strictures**

|  | Overall patients (n=200) | Anastomotic strictures (n=132) | Non-anastomotic strictures (n=68) | *p*-value |
| --- | --- | --- | --- | --- |
| Sex (male/%) | 151 (75.5) | 102 (67.5) | 49 (32.5) | 0.42 |
| Age at diagnosis, (years) mean ± SD | 52 ± 12 | 53 ± 11 | 52 ± 12 | 0.59 |
| Patients with post-ERCP cholangitis, n (%) | 92 (46) | 52 (38.6) | 40 (60.3) | **0.004** |
| Immunosuppressive therapy |  |  |  |  |
| Tacrolimus, n (%) | 107 (53.5) | 69 (52.3) | 38 (55.9) | 0.85 |
| Cyclosporin, n (%) | 74 (37) | 45 (34.1) | 29 (42.6) | 0.79 |
| Everolimus, n (%) | 5 (2.5) | 4 (3) | 1 (1.5) | 0.1 |
| Sirolimus, n (%) | 4 (2) | 2 (1.5) | 2 (2.9) | 0.7 |
| Steroids, n (%) | 36 (18) | 17 (12.9) | 19 (27.9) | 0.05 |
| MMF, n (%) | 106 (53) | 67 (50.8) | 39 (57.4) | 0.24 |
| Combined immunosuppressive therapy, n (%) | 122 (61) | 69 (52.3) | 53 (77.9) | 0.17 |
| Peri-interventional antibiotic prophylaxis, n (%) | 192 (96) | 127 (96.2) | 65 (96.2) | 0.83 |
| Microbiological analysis of the bile fluid |  |  |  |  |
| Bacteria, n (%) | 92 (46) | 52 (39.4) | 40 (58.8) | **0.009** |
| Fungi, n (%) | 27 (13.5) | 7 (5.3) | 20 (29.4) | **< 0.001** |
| Multiresistant bacteria, n (%) | 17 (18.1) | 8 (6.1) | 9 (13.2) | 0.29 |
| History of bacteria- or fungi-positive bile, n (%) | 95 (47.5) | 63 (47.7) | 32 (47.1) | 0.93 |
| ICU treatment, n (%) | 8 (4) | 6 (4.5) | 2 (2.9) | 0.33 |
| Median hospitalization duration, days [IQR] | 3 [2–7] | 3 [2–6] | 6 [2–11] | 0.96 |
| First ERCP with sphincterotomy, n (%) | 23 (11.5) | 17 (12.9) | 6 (8.8) | 0.48 |
| Repeated ERCP, n (%) | 178 (89) | 118 (89.4) | 60 (88.2) | 0.48 |
| Endoscopic intervention before cholangitis |  |  |  |  |
| Balloon dilatation, n (%) | 148 (74) | 99 (75) | 49 (72.1) | 0.87 |
| Plastic stent, n (%) | 32 (16) | 18 (13.6) | 14 (20.6) | 0.38 |
| SEMS placement, n (%) | 11 (5.5) | 9 (6.8) | 2 (2.9) | 0.07 |
| Rendezvous procedure, n (%) | 6 (3) | 3 (2.3) | 3 (4.4) | 0.7 |
| Sustained clinical success, n (%) | 150 (75) | 110 (83.3) | 40 (58.8) | **< 0.001** |
| Median time interval to sustained success, months, [IQR] | 9 [6–13] | 8 [6–12] | 10 [6–17] | **0.001** |
| Dropout before achievement of any endpoint, n (%) | 13 (6.5) | 13 (9.8) | 0 (0) |  |
| Treatment failure, n (%) | 37 (18.5) | 9 (6.8) | 28 (41.2) | **< 0.001** |
| Recurrence of biliary strictures, n (%) | 37 (18.5) | 20 (15.2) | 17 (25) | 0.09 |
| Median time interval to recurrence, months | 7 [3–16.25] | 10 [6–17] | 5 [3–13] | 0.31 |
| Successful treatment of recurrent stricture, n (%) | 29 (76.3) | 16 (12.1) | 13 (19.1) | 0.93 |
| Median number of endoscopic interventions, n [IQR] | 4 [3–6] | 4 [3–5] | 6 [4–10] | **< 0.001** |
| Re-transplantation, n (%) | 23 (11.5) | 10 (7.6) | 13 (19.1) | **0.02** |
| Death, n (%) | 78 (39) | 46 (34.8) | 32 (47.1) | 0.09 |

SD, standard deviation; IQR, interquartile range; MMF, mycophenolate mofetil; ICU, intensive care unit; ERCP, endoscopic retrograde cholangiopancreatography; SEMS, self-expanding metal stent.

Bold values indicate significant *p*-values (< 0.05).

**Supplementary Table 3: Cox regression analysis.**

| **Risk factor** |  | |
| --- | --- | --- |
|  | OR (95% CI) | *p*-value |
| Post-ERCP cholangitis | 1.19 (0.77–1.84) | 0.44 |
| Non-anastomotic stricture | 1.58 (1.01–2.42) | **0.04** |

CI, confidence interval; ERCP, endoscopic retrograde cholangiopancreatography; OR: odds ratio.

Bold values indicate significant *p* -values (< 0.05).
